# 2-Ethyl­sulfanyl-7-fluoro-3-(1*H*-1,2,4-triazol-1-yl)-4*H*-thio­chromen-4-one

**DOI:** 10.1107/S1600536810030175

**Published:** 2010-08-04

**Authors:** Tao Xiao, Yang Li, Dong-liang Liu, Guang-yan Yu

**Affiliations:** a Department of Applied Chemistry, College of Science, Nanjing University of Technology, Nanjing 210009, People’s Republic of China

## Abstract

The asymmetric unit of the title compound, C_13_H_10_FN_3_OS_2_, contains two independent mol­ecules, which differ slightly in the relative orientations of the triazole and ethyl­sulfanyl groups with respect to the planar thio­chromen-4-one frameworks. The dihedral angles between the mean planes of the triazole groups and the corresponding six-membered C_5_OS rings are 56.8 (1) and 52.9 (1)°, while the S—C—S—C dihedral angles are −11.7 (2) and −16.3 (2)°. In the crystal structure, inter­molecular C—H⋯O and C—H⋯N hydrogen bonds link the mol­ecules in a stacked arrangement along the *a* axis. A weak intramolecular C—H⋯·O interaction results in the formation of a non-planar five-membered ring.

## Related literature

For related compounds containing the 4*H*-thio­chromen-4-one fragment, see: Adams *et al.* (1991 ▶); Nakazumi *et al.* (1992 ▶); Weiss *et al.* (2008 ▶); Li, Xiao, Liu & Yu (2010 ▶); Li, Xiao, Yu & Liu (2010 ▶). For bond-length data, see: Allen *et al.* (1987 ▶).
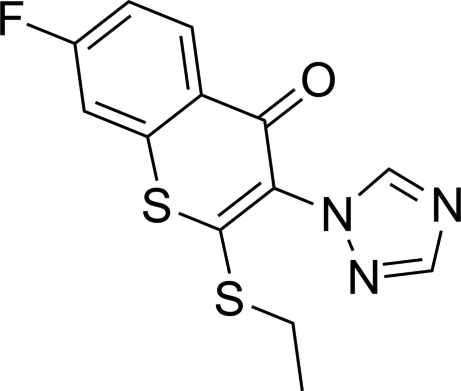


## Experimental

### 

#### Crystal data

C_13_H_10_FN_3_OS_2_
*M* *_r_* = 307.36
Triclinic, 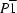

*a* = 8.1060 (16) Å
*b* = 11.288 (2) Å
*c* = 15.163 (3) Å
α = 83.12 (3)°
β = 83.15 (3)°
γ = 79.36 (3)°
*V* = 1347.0 (5) Å^3^
*Z* = 4
Mo *K*α radiation
μ = 0.41 mm^−1^
*T* = 293 K
0.30 × 0.20 × 0.10 mm

#### Data collection

Enraf–Nonius CAD-4 diffractometer
Absorption correction: ψ scan (North *et al.*, 1968 ▶) *T*_min_ = 0.888, *T*_max_ = 0.961
5271 measured reflections
4892 independent reflections
3725 reflections with *I* > 2σ(*I*)
*R*_int_ = 0.020
3 standard reflections every 200 reflections  intensity decay: 1%

#### Refinement

*R*[*F* ^2^ > 2σ(*F* ^2^)] = 0.044
*wR*(*F* ^2^) = 0.135
*S* = 1.00
4892 reflections
361 parameters
H-atom parameters constrained
Δρ_max_ = 0.21 e Å^−3^
Δρ_min_ = −0.29 e Å^−3^

### 

Data collection: *CAD-4 EXPRESS* (Enraf–Nonius, 1994 ▶); cell refinement: *CAD-4 EXPRESS*; data reduction: *XCAD4* (Harms & Wocadlo, 1995 ▶); program(s) used to solve structure: *SHELXS97* (Sheldrick, 2008 ▶); program(s) used to refine structure: *SHELXL97* (Sheldrick, 2008 ▶); molecular graphics: *PLATON* (Spek, 2009 ▶); software used to prepare material for publication: *SHELXTL* (Sheldrick, 2008 ▶).

## Supplementary Material

Crystal structure: contains datablocks I, global. DOI: 10.1107/S1600536810030175/zq2051sup1.cif

Structure factors: contains datablocks I. DOI: 10.1107/S1600536810030175/zq2051Isup2.hkl

Additional supplementary materials:  crystallographic information; 3D view; checkCIF report

## Figures and Tables

**Table 1:** Hydrogen-bond geometry (Å, °)

*D*—H⋯*A*	*D*—H	H⋯*A*	*D*⋯*A*	*D*—H⋯*A*
C5—H5*A*⋯O2^i^	0.93	2.40	3.231 (4)	149
C8—H8*A*⋯O1	0.93	2.45	2.764 (4)	100
C8—H8*A*⋯N6^ii^	0.93	2.56	3.381 (4)	147
C18—H18*A*⋯O1^iii^	0.93	2.51	3.334 (4)	147
C21—H21*A*⋯O2	0.93	2.46	2.772 (4)	100
C21—H21*A*⋯N3^iv^	0.93	2.47	3.326 (4)	154

Symmetry codes: (i) 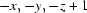; (ii) 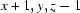; (iii) 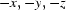; (iv) 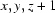.

**Fig. 1.**
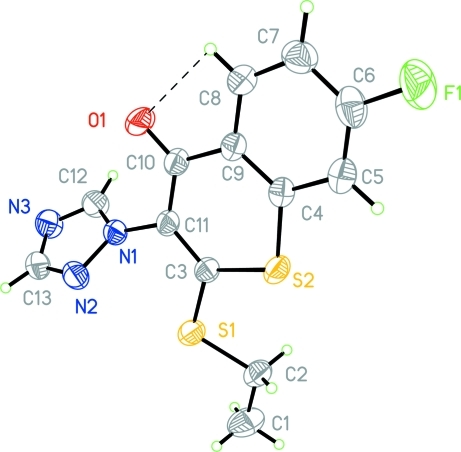
The molecular structure of (I) with the atom-numbering scheme. Displacement ellipsoids are drawn at the 50% probability level.

**Fig. 2.**
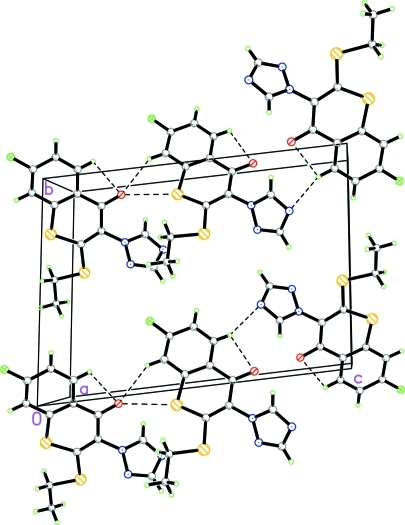
A packing diagram of (I). The shortest interactions are shown as dashed lines.

## supplementary crystallographic information

### Comment

The title compound, C_13_H_10_ON_3_S_2_F, is a new molecule which has a potential use as antifungal. Its molecular structure of is shown in Fig. 1 and selected geometric parameters are given in Table 1. The bond lengths and angles are within normal ranges (Allen *et al.*, 1987). The asymmetric unit of the title compound contains two independent molecules. They differ slightly in the relative orientations of the triazole and ethylsulfanyl groups with respect to the planar thiochromen-4-one frameworks. The dihedral angles between the mean planes of the triazole groups and the corresponding six-membered C_5_OS rings are 56.8 (1) and 52.9 (1)° while the dihedral angles S2-C3-C1-C2 and S4-C16-S3-C15 are -11.7 (2) and -16.3 (2)°. In the crystal structure, intermolecular C—H···O and C—H···N hydrogen bonds (Table 2) link the molecules in a stacked arrangement along the *a* axis (Fig. 2).

### Experimental

CS2 (2.0 g, 26.3 mmol) was dropwise added to a solution of 1-(2,4- difluorophenyl)-2-(1*H*-1,2,4-triazol-1-yl) ethanone (5 g, 22.4 mmol) in DMSO (20 ml) containing NaOH (1.8 g, 45 mmol). The yellow solution was stirred for about 2 h at room temperature. Then bromethyl (2.5 g, 22.4 mmol) was dropwise added to the intermediate. After 3 h, the solution was poured into water (50 ml). The crystalline product was isolated by filtration, washed with water (300 ml). The crystals were obtained by dissolving the product in acetone (20 ml) and evaporating acetone slowly at room temperature for about 7 d.

### Refinement

The H atoms were positioned geometrically with C—H = 0.93 Å for aromatic H atoms, C—H = 0.97 Å for methylene H atoms, and with C—H = 0.96 Å for methyl H atoms, and constrained to ride on their parent atoms, with *U*_iso_(H) = *xU*_eq_(C/O), where *x* = 1.2 for aromatic and methylene H atoms and *x* = 1.5 for the other H atoms.

### Crystal data

**Table:**

C_13_H_10_FN_3_OS_2_	*Z* = 4
*M**_r_* = 307.36	*F*(000) = 632
Triclinic, *P*1	*D*_x_ = 1.516 Mg m^−^^3^
Hall symbol: -P 1	Melting point: 432 K
*a* = 8.1060 (16) Å	Mo *K*α radiation, λ = 0.71073 Å
*b* = 11.288 (2) Å	Cell parameters from 25 reflections
*c* = 15.163 (3) Å	θ = 9–14°
α = 83.12 (3)°	µ = 0.41 mm^−^^1^
β = 83.15 (3)°	*T* = 293 K
γ = 79.36 (3)°	Block, yellow
*V* = 1347.0 (5) Å^3^	0.30 × 0.20 × 0.10 mm

### Data collection

**Table:**

Enraf–Nonius CAD-4 diffractometer	3725 reflections with *I* > 2σ(*I*)
Radiation source: fine-focus sealed tube	*R*_int_ = 0.020
graphite	θ_max_ = 25.3°, θ_min_ = 1.4°
ω/2θ scans	*h* = 0→9
Absorption correction: ψ scan (North *et al.*, 1968)	*k* = −13→13
*T*_min_ = 0.888, *T*_max_ = 0.961	*l* = −18→18
5271 measured reflections	3 standard reflections every 200 reflections
4892 independent reflections	intensity decay: 1%

### Refinement

**Table:**

Refinement on *F*^2^	Primary atom site location: structure-invariant direct methods
Least-squares matrix: full	Secondary atom site location: difference Fourier map
*R*[*F*^2^ > 2σ(*F*^2^)] = 0.044	Hydrogen site location: inferred from neighbouring sites
*wR*(*F*^2^) = 0.135	H-atom parameters constrained
*S* = 1.00	*w* = 1/[σ^2^(*F*_o_^2^) + (0.087*P*)^2^] where *P* = (*F*_o_^2^ + 2*F*_c_^2^)/3
4892 reflections	(Δ/σ)_max_ < 0.001
361 parameters	Δρ_max_ = 0.21 e Å^−^^3^
0 restraints	Δρ_min_ = −0.29 e Å^−^^3^

### Special details

**Table:**

Geometry. All e.s.d.'s (except the e.s.d. in the dihedral angle between two l.s. planes) are estimated using the full covariance matrix. The cell e.s.d.'s are taken into account individually in the estimation of e.s.d.'s in distances, angles and torsion angles; correlations between e.s.d.'s in cell parameters are only used when they are defined by crystal symmetry. An approximate (isotropic) treatment of cell e.s.d.'s is used for estimating e.s.d.'s involving l.s. planes.
Refinement. Refinement of *F*^2^ against ALL reflections. The weighted *R*-factor *wR* and goodness of fit *S* are based on *F*^2^, conventional *R*-factors *R* are based on *F*, with *F* set to zero for negative *F*^2^. The threshold expression of *F*^2^ > σ(*F*^2^) is used only for calculating *R*-factors(gt) *etc*. and is not relevant to the choice of reflections for refinement. *R*-factors based on *F*^2^ are statistically about twice as large as those based on *F*, and *R*- factors based on ALL data will be even larger.

### Fractional atomic coordinates and isotropic or equivalent isotropic displacement parameters (Å^2^)

**Table:**

	*x*	*y*	*z*	*U*_iso_*/*U*_eq_	
S1	−0.15082 (10)	0.40138 (7)	−0.02746 (5)	0.0448 (2)	
S2	0.05140 (10)	0.20004 (7)	0.07527 (4)	0.0415 (2)	
F1	0.4481 (3)	−0.17048 (19)	0.17733 (13)	0.0733 (6)	
O1	0.1829 (3)	0.05374 (19)	−0.18751 (13)	0.0564 (6)	
N1	−0.0026 (3)	0.2800 (2)	−0.18726 (14)	0.0374 (5)	
N2	0.0365 (4)	0.3935 (2)	−0.21190 (16)	0.0474 (6)	
N3	−0.0921 (4)	0.3345 (2)	−0.31908 (16)	0.0505 (7)	
C1	−0.3454 (5)	0.5338 (3)	0.0936 (2)	0.0671 (10)	
H1A	−0.3849	0.5452	0.1547	0.101*	
H1B	−0.4391	0.5298	0.0615	0.101*	
H1C	−0.2920	0.6005	0.0671	0.101*	
C2	−0.2196 (4)	0.4170 (3)	0.08944 (19)	0.0483 (8)	
H2B	−0.2719	0.3488	0.1161	0.058*	
H2C	−0.1242	0.4199	0.1216	0.058*	
C3	−0.0111 (3)	0.2636 (2)	−0.02745 (17)	0.0346 (6)	
C4	0.1860 (3)	0.0661 (2)	0.05066 (17)	0.0335 (6)	
C5	0.2616 (4)	−0.0010 (3)	0.12351 (19)	0.0423 (7)	
H5A	0.2386	0.0250	0.1803	0.051*	
C6	0.3705 (4)	−0.1061 (3)	0.1084 (2)	0.0489 (8)	
C7	0.4072 (4)	−0.1493 (3)	0.0251 (2)	0.0476 (7)	
H7A	0.4816	−0.2214	0.0173	0.057*	
C8	0.3312 (4)	−0.0832 (3)	−0.04493 (19)	0.0419 (7)	
H8A	0.3534	−0.1116	−0.1010	0.050*	
C9	0.2199 (3)	0.0267 (2)	−0.03439 (17)	0.0335 (6)	
C10	0.1509 (4)	0.0959 (2)	−0.11443 (17)	0.0369 (6)	
C11	0.0459 (3)	0.2127 (2)	−0.10583 (17)	0.0340 (6)	
C12	−0.0763 (4)	0.2477 (3)	−0.25322 (19)	0.0460 (7)	
H12A	−0.1115	0.1737	−0.2524	0.055*	
C13	−0.0206 (4)	0.4192 (3)	−0.29026 (19)	0.0485 (8)	
H13A	−0.0122	0.4924	−0.3244	0.058*	
S3	−0.37421 (11)	−0.30227 (7)	0.49425 (5)	0.0502 (2)	
S4	−0.26104 (10)	−0.08916 (6)	0.39414 (4)	0.0418 (2)	
F2	−0.1135 (3)	0.31666 (19)	0.28874 (13)	0.0754 (7)	
O2	−0.2316 (3)	0.0220 (2)	0.66100 (13)	0.0554 (6)	
N4	−0.3399 (3)	−0.1897 (2)	0.65791 (14)	0.0391 (5)	
N5	−0.2627 (4)	−0.3070 (2)	0.67609 (17)	0.0596 (8)	
N6	−0.4426 (4)	−0.2446 (2)	0.79373 (16)	0.0547 (7)	
C14	−0.4777 (5)	−0.4194 (3)	0.3729 (3)	0.0699 (11)	
H14A	−0.5044	−0.4217	0.3133	0.105*	
H14B	−0.5756	−0.4267	0.4141	0.105*	
H14C	−0.3883	−0.4852	0.3869	0.105*	
C15	−0.4220 (4)	−0.3008 (3)	0.3800 (2)	0.0519 (8)	
H15A	−0.5110	−0.2335	0.3658	0.062*	
H15B	−0.3227	−0.2924	0.3388	0.062*	
C16	−0.3121 (3)	−0.1627 (2)	0.49766 (17)	0.0367 (6)	
C17	−0.2133 (3)	0.0464 (2)	0.41911 (17)	0.0355 (6)	
C18	−0.1784 (4)	0.1276 (3)	0.34501 (19)	0.0441 (7)	
H18A	−0.1813	0.1085	0.2873	0.053*	
C19	−0.1398 (4)	0.2359 (3)	0.3600 (2)	0.0487 (8)	
C20	−0.1302 (4)	0.2667 (3)	0.4445 (2)	0.0492 (8)	
H20A	−0.1024	0.3408	0.4524	0.059*	
C21	−0.1624 (4)	0.1861 (3)	0.51620 (19)	0.0427 (7)	
H21A	−0.1550	0.2054	0.5734	0.051*	
C22	−0.2065 (3)	0.0748 (2)	0.50541 (17)	0.0359 (6)	
C23	−0.2438 (4)	−0.0071 (3)	0.58617 (17)	0.0383 (6)	
C24	−0.2987 (3)	−0.1197 (2)	0.57607 (17)	0.0359 (6)	
C25	−0.4443 (4)	−0.1566 (3)	0.72971 (19)	0.0439 (7)	
H25A	−0.5098	−0.0801	0.7333	0.053*	
C26	−0.3311 (5)	−0.3339 (3)	0.7569 (2)	0.0652 (10)	
H26A	−0.3043	−0.4105	0.7873	0.078*	

### Atomic displacement parameters (Å^2^)

**Table:**

	*U*^11^	*U*^22^	*U*^33^	*U*^12^	*U*^13^	*U*^23^
S1	0.0554 (5)	0.0408 (4)	0.0355 (4)	0.0018 (3)	−0.0064 (3)	−0.0074 (3)
S2	0.0526 (5)	0.0448 (4)	0.0255 (3)	0.0000 (3)	−0.0058 (3)	−0.0078 (3)
F1	0.0731 (14)	0.0811 (14)	0.0548 (12)	0.0135 (11)	−0.0248 (11)	0.0130 (10)
O1	0.0879 (17)	0.0500 (12)	0.0260 (10)	0.0068 (12)	−0.0063 (11)	−0.0105 (9)
N1	0.0508 (14)	0.0344 (12)	0.0284 (11)	−0.0104 (11)	−0.0063 (10)	−0.0021 (9)
N2	0.0697 (18)	0.0384 (13)	0.0372 (13)	−0.0180 (12)	−0.0082 (12)	0.0000 (10)
N3	0.0672 (18)	0.0487 (15)	0.0369 (13)	−0.0085 (13)	−0.0183 (13)	0.0007 (11)
C1	0.062 (2)	0.067 (2)	0.070 (2)	0.0081 (19)	−0.0055 (19)	−0.0280 (19)
C2	0.0549 (19)	0.0489 (17)	0.0391 (16)	−0.0026 (15)	0.0006 (14)	−0.0117 (13)
C3	0.0408 (15)	0.0357 (14)	0.0293 (13)	−0.0092 (12)	−0.0068 (12)	−0.0049 (11)
C4	0.0333 (14)	0.0389 (15)	0.0297 (13)	−0.0108 (12)	−0.0036 (11)	−0.0016 (11)
C5	0.0457 (17)	0.0504 (17)	0.0317 (15)	−0.0112 (14)	−0.0054 (13)	−0.0013 (13)
C6	0.0417 (17)	0.0577 (19)	0.0442 (18)	−0.0061 (15)	−0.0110 (14)	0.0104 (15)
C7	0.0420 (17)	0.0426 (17)	0.0541 (19)	−0.0012 (14)	−0.0004 (15)	−0.0018 (14)
C8	0.0465 (17)	0.0412 (16)	0.0366 (15)	−0.0066 (13)	0.0025 (13)	−0.0058 (12)
C9	0.0340 (14)	0.0377 (14)	0.0294 (13)	−0.0090 (12)	−0.0005 (11)	−0.0039 (11)
C10	0.0431 (16)	0.0393 (15)	0.0287 (14)	−0.0089 (13)	−0.0013 (12)	−0.0044 (11)
C11	0.0430 (16)	0.0354 (14)	0.0256 (13)	−0.0106 (12)	−0.0048 (11)	−0.0031 (11)
C12	0.060 (2)	0.0435 (16)	0.0386 (16)	−0.0134 (15)	−0.0140 (14)	−0.0045 (13)
C13	0.067 (2)	0.0403 (16)	0.0354 (16)	−0.0082 (15)	−0.0043 (15)	0.0032 (13)
S3	0.0691 (6)	0.0424 (4)	0.0421 (4)	−0.0165 (4)	−0.0088 (4)	−0.0015 (3)
S4	0.0586 (5)	0.0421 (4)	0.0258 (4)	−0.0112 (3)	−0.0035 (3)	−0.0048 (3)
F2	0.1168 (19)	0.0736 (14)	0.0465 (11)	−0.0540 (13)	−0.0166 (11)	0.0177 (10)
O2	0.0827 (17)	0.0599 (14)	0.0287 (11)	−0.0237 (12)	−0.0071 (11)	−0.0062 (9)
N4	0.0420 (14)	0.0436 (13)	0.0289 (12)	−0.0032 (11)	−0.0011 (10)	−0.0009 (10)
N5	0.073 (2)	0.0467 (15)	0.0439 (15)	0.0110 (14)	0.0090 (14)	0.0074 (12)
N6	0.0624 (18)	0.0575 (17)	0.0366 (14)	−0.0042 (14)	0.0075 (13)	0.0038 (12)
C14	0.087 (3)	0.064 (2)	0.068 (2)	−0.023 (2)	−0.015 (2)	−0.0210 (19)
C15	0.061 (2)	0.0527 (19)	0.0441 (18)	−0.0092 (16)	−0.0056 (16)	−0.0123 (15)
C16	0.0371 (15)	0.0382 (15)	0.0328 (14)	−0.0022 (12)	−0.0023 (12)	−0.0028 (11)
C17	0.0354 (15)	0.0426 (15)	0.0276 (13)	−0.0050 (12)	−0.0030 (11)	−0.0028 (11)
C18	0.0482 (18)	0.0550 (18)	0.0309 (15)	−0.0136 (15)	−0.0058 (13)	−0.0023 (13)
C19	0.0531 (19)	0.0575 (19)	0.0384 (16)	−0.0210 (16)	−0.0079 (14)	0.0052 (14)
C20	0.054 (2)	0.0489 (18)	0.0495 (18)	−0.0194 (15)	−0.0093 (15)	−0.0039 (14)
C21	0.0459 (17)	0.0492 (17)	0.0353 (15)	−0.0101 (14)	−0.0077 (13)	−0.0073 (13)
C22	0.0338 (15)	0.0408 (15)	0.0332 (14)	−0.0049 (12)	−0.0063 (12)	−0.0031 (12)
C23	0.0423 (16)	0.0433 (16)	0.0277 (14)	−0.0027 (13)	−0.0038 (12)	−0.0036 (12)
C24	0.0356 (15)	0.0418 (15)	0.0270 (13)	−0.0002 (12)	−0.0012 (11)	−0.0018 (11)
C25	0.0454 (17)	0.0479 (17)	0.0356 (15)	−0.0048 (14)	0.0039 (13)	−0.0060 (13)
C26	0.083 (3)	0.053 (2)	0.0447 (19)	0.0065 (19)	0.0102 (18)	0.0120 (15)

### Geometric parameters (Å, °)

**Table:**

S1—C3	1.747 (3)	S3—C16	1.749 (3)
S1—C2	1.813 (3)	S3—C15	1.817 (3)
S2—C3	1.727 (3)	S4—C16	1.727 (3)
S2—C4	1.745 (3)	S4—C17	1.740 (3)
F1—C6	1.350 (3)	F2—C19	1.351 (3)
O1—C10	1.238 (3)	O2—C23	1.238 (3)
N1—C12	1.342 (3)	N4—C25	1.343 (4)
N1—N2	1.375 (3)	N4—N5	1.367 (3)
N1—C11	1.427 (3)	N4—C24	1.428 (3)
N2—C13	1.308 (4)	N5—C26	1.308 (4)
N3—C12	1.313 (4)	N6—C25	1.303 (4)
N3—C13	1.344 (4)	N6—C26	1.350 (4)
C1—C2	1.513 (4)	C14—C15	1.509 (4)
C1—H1A	0.9600	C14—H14A	0.9600
C1—H1B	0.9600	C14—H14B	0.9600
C1—H1C	0.9600	C14—H14C	0.9600
C2—H2B	0.9700	C15—H15A	0.9700
C2—H2C	0.9700	C15—H15B	0.9700
C3—C11	1.375 (4)	C16—C24	1.360 (4)
C4—C9	1.394 (4)	C17—C22	1.393 (4)
C4—C5	1.400 (4)	C17—C18	1.398 (4)
C5—C6	1.367 (4)	C18—C19	1.368 (4)
C5—H5A	0.9300	C18—H18A	0.9300
C6—C7	1.387 (4)	C19—C20	1.381 (4)
C7—C8	1.364 (4)	C20—C21	1.364 (4)
C7—H7A	0.9300	C20—H20A	0.9300
C8—C9	1.407 (4)	C21—C22	1.401 (4)
C8—H8A	0.9300	C21—H21A	0.9300
C9—C10	1.472 (4)	C22—C23	1.479 (4)
C10—C11	1.443 (4)	C23—C24	1.452 (4)
C12—H12A	0.9300	C25—H25A	0.9300
C13—H13A	0.9300	C26—H26A	0.9300
			
C3—S1—C2	104.62 (14)	C16—S3—C15	104.65 (14)
C3—S2—C4	103.44 (13)	C16—S4—C17	103.64 (13)
C12—N1—N2	108.8 (2)	C25—N4—N5	108.5 (2)
C12—N1—C11	130.1 (2)	C25—N4—C24	129.6 (2)
N2—N1—C11	120.9 (2)	N5—N4—C24	121.8 (2)
C13—N2—N1	101.4 (2)	C26—N5—N4	101.7 (3)
C12—N3—C13	102.1 (2)	C25—N6—C26	101.7 (3)
C2—C1—H1A	109.5	C15—C14—H14A	109.5
C2—C1—H1B	109.5	C15—C14—H14B	109.5
H1A—C1—H1B	109.5	H14A—C14—H14B	109.5
C2—C1—H1C	109.5	C15—C14—H14C	109.5
H1A—C1—H1C	109.5	H14A—C14—H14C	109.5
H1B—C1—H1C	109.5	H14B—C14—H14C	109.5
C1—C2—S1	107.1 (2)	C14—C15—S3	107.2 (2)
C1—C2—H2B	110.3	C14—C15—H15A	110.3
S1—C2—H2B	110.3	S3—C15—H15A	110.3
C1—C2—H2C	110.3	C14—C15—H15B	110.3
S1—C2—H2C	110.3	S3—C15—H15B	110.3
H2B—C2—H2C	108.6	H15A—C15—H15B	108.5
C11—C3—S2	123.5 (2)	C24—C16—S4	123.6 (2)
C11—C3—S1	120.6 (2)	C24—C16—S3	121.9 (2)
S2—C3—S1	115.83 (15)	S4—C16—S3	114.44 (15)
C9—C4—C5	121.1 (3)	C22—C17—C18	120.8 (3)
C9—C4—S2	124.0 (2)	C22—C17—S4	124.2 (2)
C5—C4—S2	114.9 (2)	C18—C17—S4	115.0 (2)
C6—C5—C4	117.8 (3)	C19—C18—C17	117.9 (3)
C6—C5—H5A	121.1	C19—C18—H18A	121.0
C4—C5—H5A	121.1	C17—C18—H18A	121.0
F1—C6—C5	118.7 (3)	F2—C19—C18	118.0 (3)
F1—C6—C7	118.2 (3)	F2—C19—C20	119.1 (3)
C5—C6—C7	123.2 (3)	C18—C19—C20	122.9 (3)
C8—C7—C6	118.2 (3)	C21—C20—C19	118.6 (3)
C8—C7—H7A	120.9	C21—C20—H20A	120.7
C6—C7—H7A	120.9	C19—C20—H20A	120.7
C7—C8—C9	121.6 (3)	C20—C21—C22	121.3 (3)
C7—C8—H8A	119.2	C20—C21—H21A	119.4
C9—C8—H8A	119.2	C22—C21—H21A	119.4
C4—C9—C8	118.1 (3)	C17—C22—C21	118.4 (3)
C4—C9—C10	123.7 (2)	C17—C22—C23	123.1 (3)
C8—C9—C10	118.1 (2)	C21—C22—C23	118.5 (2)
O1—C10—C11	121.0 (2)	O2—C23—C24	121.0 (3)
O1—C10—C9	120.2 (2)	O2—C23—C22	120.0 (3)
C11—C10—C9	118.8 (2)	C24—C23—C22	118.9 (2)
C3—C11—N1	118.0 (2)	C16—C24—N4	118.9 (3)
C3—C11—C10	126.2 (2)	C16—C24—C23	126.2 (3)
N1—C11—C10	115.8 (2)	N4—C24—C23	114.9 (2)
N3—C12—N1	111.0 (3)	N6—C25—N4	111.7 (3)
N3—C12—H12A	124.5	N6—C25—H25A	124.2
N1—C12—H12A	124.5	N4—C25—H25A	124.2
N2—C13—N3	116.7 (3)	N5—C26—N6	116.4 (3)
N2—C13—H13A	121.7	N5—C26—H26A	121.8
N3—C13—H13A	121.7	N6—C26—H26A	121.8
			
C12—N1—N2—C13	1.0 (3)	C25—N4—N5—C26	1.2 (4)
C11—N1—N2—C13	176.8 (3)	C24—N4—N5—C26	177.5 (3)
C3—S1—C2—C1	−178.5 (2)	C16—S3—C15—C14	−179.7 (2)
C4—S2—C3—C11	−1.7 (3)	C17—S4—C16—C24	−4.9 (3)
C4—S2—C3—S1	179.60 (15)	C17—S4—C16—S3	177.84 (15)
C2—S1—C3—C11	169.6 (2)	C15—S3—C16—C24	166.5 (2)
C2—S1—C3—S2	−11.7 (2)	C15—S3—C16—S4	−16.2 (2)
C3—S2—C4—C9	−2.3 (3)	C16—S4—C17—C22	5.2 (3)
C3—S2—C4—C5	177.1 (2)	C16—S4—C17—C18	−175.8 (2)
C9—C4—C5—C6	0.4 (4)	C22—C17—C18—C19	−0.9 (4)
S2—C4—C5—C6	−179.1 (2)	S4—C17—C18—C19	−180.0 (2)
C4—C5—C6—F1	178.3 (3)	C17—C18—C19—F2	−177.1 (3)
C4—C5—C6—C7	−0.9 (5)	C17—C18—C19—C20	1.5 (5)
F1—C6—C7—C8	−178.9 (3)	F2—C19—C20—C21	177.9 (3)
C5—C6—C7—C8	0.3 (5)	C18—C19—C20—C21	−0.7 (5)
C6—C7—C8—C9	0.8 (5)	C19—C20—C21—C22	−0.8 (5)
C5—C4—C9—C8	0.6 (4)	C18—C17—C22—C21	−0.5 (4)
S2—C4—C9—C8	180.0 (2)	S4—C17—C22—C21	178.5 (2)
C5—C4—C9—C10	−177.0 (3)	C18—C17—C22—C23	179.4 (3)
S2—C4—C9—C10	2.4 (4)	S4—C17—C22—C23	−1.6 (4)
C7—C8—C9—C4	−1.2 (4)	C20—C21—C22—C17	1.4 (4)
C7—C8—C9—C10	176.5 (3)	C20—C21—C22—C23	−178.5 (3)
C4—C9—C10—O1	−178.1 (3)	C17—C22—C23—O2	178.3 (3)
C8—C9—C10—O1	4.3 (4)	C21—C22—C23—O2	−1.8 (4)
C4—C9—C10—C11	1.8 (4)	C17—C22—C23—C24	−3.3 (4)
C8—C9—C10—C11	−175.8 (2)	C21—C22—C23—C24	176.6 (2)
S2—C3—C11—N1	−174.04 (19)	S4—C16—C24—N4	−178.25 (19)
S1—C3—C11—N1	4.6 (4)	S3—C16—C24—N4	−1.2 (4)
S2—C3—C11—C10	6.3 (4)	S4—C16—C24—C23	1.1 (4)
S1—C3—C11—C10	−175.1 (2)	S3—C16—C24—C23	178.1 (2)
C12—N1—C11—C3	−126.8 (3)	C25—N4—C24—C16	−128.4 (3)
N2—N1—C11—C3	58.4 (4)	N5—N4—C24—C16	56.1 (4)
C12—N1—C11—C10	52.9 (4)	C25—N4—C24—C23	52.2 (4)
N2—N1—C11—C10	−121.8 (3)	N5—N4—C24—C23	−123.3 (3)
O1—C10—C11—C3	173.5 (3)	O2—C23—C24—C16	−177.9 (3)
C9—C10—C11—C3	−6.4 (4)	C22—C23—C24—C16	3.7 (4)
O1—C10—C11—N1	−6.2 (4)	O2—C23—C24—N4	1.4 (4)
C9—C10—C11—N1	173.9 (2)	C22—C23—C24—N4	−177.0 (2)
C13—N3—C12—N1	1.4 (4)	C26—N6—C25—N4	−0.1 (4)
N2—N1—C12—N3	−1.6 (4)	N5—N4—C25—N6	−0.7 (4)
C11—N1—C12—N3	−176.8 (3)	C24—N4—C25—N6	−176.7 (3)
N1—N2—C13—N3	−0.1 (4)	N4—N5—C26—N6	−1.4 (5)
C12—N3—C13—N2	−0.8 (4)	C25—N6—C26—N5	1.0 (5)

### Hydrogen-bond geometry (Å, °)

**Table:**

*D*—H···*A*	*D*—H	H···*A*	*D*···*A*	*D*—H···*A*
C5—H5A···O2^i^	0.93	2.40	3.231 (4)	149.
C8—H8A···O1	0.93	2.45	2.764 (4)	100.
C8—H8A···N6^ii^	0.93	2.56	3.381 (4)	147.
C18—H18A···O1^iii^	0.93	2.51	3.334 (4)	147.
C21—H21A···O2	0.93	2.46	2.772 (4)	100.
C21—H21A···N3^iv^	0.93	2.47	3.326 (4)	154.

Symmetry codes: (i) −*x*, −*y*, −*z*+1; (ii) *x*+1, *y*, *z*−1; (iii) −*x*, −*y*, −*z*; (iv) *x*, *y*, *z*+1.

## References

[bb1] Adams, H., Bailey, N. A., Giles, P. R. & Marson, C. M. (1991). *Acta Cryst.* C**47**, 1332–1334.

[bb2] Allen, F. H., Kennard, O., Watson, D. G., Brammer, L., Orpen, A. G. & Taylor, R. (1987). *J. Chem. Soc. Perkin Trans. 2*, pp. S1–19.

[bb3] Enraf–Nonius (1994). *CAD-4 EXPRESS* Enraf–Nonius, Delft, The Netherlands.

[bb4] Harms, K. & Wocadlo, S. (1995). *XCAD4* University of Marburg, Germany.

[bb5] Li, Y., Xiao, T., Liu, D. & Yu, G. (2010). *Acta Cryst.* E**66**, o694. 10.1107/S1600536810002667 PMC2983649 21580435

[bb6] Li, Y., Xiao, T., Yu, G. & Liu, D. (2010). *Acta Cryst.* E**66**, o2072. 10.1107/S1600536810027467 PMC3007513 21588373

[bb7] Nakazumi, H., Watanabe, S. & Kitao, T. (1992). *J. Chem. Res.* **212**, 1616–1641.

[bb8] North, A. C. T., Phillips, D. C. & Mathews, F. S. (1968). *Acta Cryst.* A**24**, 351–359.

[bb9] Sheldrick, G. M. (2008). *Acta Cryst.* A**64**, 112–122. 10.1107/S0108767307043930 18156677

[bb10] Spek, A. L. (2009). *Acta Cryst.* D**65**, 148–155. 10.1107/S090744490804362X PMC2631630 19171970

[bb11] Weiss, R., Bess, M., Huber, S. M. & Heinemann, F. W. (2008). *J. Am. Chem. Soc.* **130**, 4610–4617. 10.1021/ja071316a 18341332

